# 

**Published:** 1893-05-27

**Authors:** 


### CONTENTS.

**Huxley's Logic and Life** - 129
**What the Hospitals do for the Public.** By Sir William S. Savory, Bart, F.R.S. - 130
**Medical History from the Earliest Times.** By E. T. Withington, M.A., M.B.Oxon. LVI.—The Iatromechanical School - 131
**Contemporary Theory and Research** - 132
**How to Keep Well in Africa.** II.—Cure - 133
**Gleanings in Science and Medicine** - 134
**Annotations.**—"Washed Daily on the Allotment System"—Is Cancer Increasing?—George Smith: The Man or his Monument? 135
**Notes and News** - 136
**New Drugs and Preparations.**—Salophen - 140
**Scraps and Gleanings** - 144
**The Hospital Clinic**—Sussex County Hospital.—Treatment of his Simple Fracture of the Tibia and Fibula - 137
Rhinoscopy. III.—Examination of the Nose - 138
New Appliances and Things Medical.—Digestive Agents and Concentrated Food Stuffs - 139
**The Institutional Workshop**—Within the Hospitals.—Convalescent Home for Charing Cross Hospital - 141
Practical Departments.—Reading Stands, &c. - 142
The Story of the Insane from Year to Year - 142
Construction Notes - 143
Festival Dinners and Meetings.—The Great Northern Central Hospital—Hospital for Women, Soho Square - 144
**The Book World of Medicine and Science** - vii
**Medical Vacancies and Appointments** - x

### EXTRA SUPPLEMENT.—THE NURSING MIRROR.

**News from the Nursing World.**—The Nurse Dolls—False Economy—The Worth of a Finger—Does Massage Pay?—The Plague of Flies—Save Me from My Friends—Ladies' Sanitary Association—Trains and Trained Nurses—A Good Example—Nursing at Hinckley—From Church to Ward—District Nursing at Yarmouth—Rio Janeiro—Short Items—"The Hospital" Endowed Bed - lxxxi

**Sanitation for Nurses.** By A Sanitary Inspector. VII.—Notification in Foreign Countries—Finland - lxxxiii

**The Leper Asylum, Colombo, Ceylon.** By a Colombo Resident - lxxxiv

**Nursing in India.**—The Dufferin Hospital, Allahabad - lxxxiv

**London Hospital.**—Students' Concert - lxxxv

**Novelties for Nurses.**—Boots, Shoes, and Cosy Teas - lxxxv

**Everybody's Opinion.** Measles—Fever Nursing - lxxxv

**Spain—Where to Go** - lxxxv

**Nursing in the Land of Ophir.**—The Hospital at Umtali lxxxvi

**The Royal British Nurses' Association** - lxxxvi

**For Reading to the Sick.**—"Whims" - lxxxvii

**A Neglected Field—Notes and Queries** - lxxxvii

**The Muses' Looking Glass** - lxxxviii

**The Book World for Women and Nurses** - lxxxix

